# Diversity of HIV-1 genotypes and high prevalence of pretreatment drug resistance in newly diagnosed HIV-infected patients in Shanghai, China

**DOI:** 10.1186/s12879-019-3927-1

**Published:** 2019-04-08

**Authors:** Zhenyan Wang, Min Zhang, Renfang Zhang, Li Liu, Yinzhong Shen, Jiangrong Wang, Hongzhou Lu

**Affiliations:** 1 Fudan University (Shanghai Public Health Clinical Center), Shanghai, China; 2 Clinical laboratory, Shanghai Public Health Clinical Center, Shanghai, China; 3 Department of infections and immunity, Shanghai Public Health Clinical Center, 2901 Caolang Road, Jinshan District, Shanghai, 201508 China; 4 Department of infectious diseases, Huashan Hospital affiliated to Fudan University, Shanghai, China

**Keywords:** HIV-1, AIDS, Genotype, Pretreatment drug resistance, Mutation

## Abstract

**Background:**

Genetic variability and liability to develop drug-resistant mutations are the main characteristics of HIV-1, which can not only increase the risk of antiretroviral treatment (ART) failure, but also can lead to the spread of resistant strains. We aim to investigate the distribution of HIV-1 genotypes and prevalence of pretreatment drug resistance (PDR) in ART-naïve HIV-1 infected patients in Shanghai China.

**Methods:**

A cross-sectional study was performed among the newly diagnosed ART-naive HIV-1 infected patients during the period from January 2017 to November 2017 in Shanghai Public Health Clinical Center. The target fragment of 1316 bp in the *pol* gene spanning the reverse transcriptase and protease regions was amplified using a nested polymerase chain reaction. HIV-1 genotypes were determined by phylogenetic analysis, and PDR associated mutations were determined according to Stanford University HIV Drug Resistance Database (http://hivdb.stanford.edu/).

**Results:**

We successfully amplified *pol* gene sequences from blood samples of 317 patients, of whom 95.3% were male, and 68.8% were men who have sex with men. The median age was 33 years; and the median CD4 count was 275 cells/μL. The predominant HIV-1 genotype was circulating recombinant form (CRF) 01_AE (53.0%, 168/317), followed by CRF07_BC (29.7%, 94/317), B (7.6%, 24/317), CRF08_BC (1.9%, 6/317), CRF55_01B (1.9%, 6/317), CRF 59_01B (0.9%, 3/317). In addition, 5% (16/317) HIV-1 strains were identified as other subtypes or CRFs/URFs (unique recombinant forms). The overall prevalence of PDR was 17.4% (55/317). PDR frequency to non-nucleoside reverse transcriptase inhibitor (NNRTI, 16.4%) was much higher than that to nucleoside reverse transcriptase inhibitor (NRTI, 4.7%) and protease inhibitor (PI, 0.6%). The most common HIV-1 mutation pattern for NNRTI and NRTI were V179D/E (10.1%, 32/317) and M184 V (2.8%, 9/317), respectively. About half (49.1%, 27/55) of the HIV-1 strains with mutation presented as potential low-level resistant to NNRTI attributed to V179D/E.

**Conclusion:**

The distribution of HIV-1 genotypes in Shanghai China is diverse and complex. The high prevalence of PDR highlights the significance of baseline HIV-1 drug resistance testing. Non-NNRTI-containing regimen may be the preferred initial therapy for newly diagnosed HIV-1 patients in Shanghai in the absence of PDR test results.

## Background

HIV/AIDS remains a major public health problem in China. According to the official report, since the first HIV-infected case found in 1985, the number of people living with HIV and deaths due to HIV/AIDS had reached to 83,1225 and 255,995 by the end of July 2018, respectively. Furthermore, the number of new HIV infections continue to rise with a total of 134, 512 reported in 2017 [1, 2].

The main characteristics of HIV are its enormous genetic variability and liability to develop drug-resistant mutations along with high rates of virus replication. Based on the phylogenetic analysis data, HIV can be divided into two major types, HIV type 1 (HIV-1) and HIV type 2 (HIV-2). HIV-1 is further classified into four distinct groups: M (major), O(outlier), N (non-M, non-O), and P (pending) [3]. Group M viruses are responsible for the AIDS pandemic globally, and nine subtypes, designated by the letters A-D, F-H, J and K, are recognized within this group [4]. Recombinants between subtypes have been designated as circulating recombinant forms (CRFs) if fully sequenced and found in three or more epidemiologically unlinked individuals, and unique recombinant forms (URFs) if not meeting these criteria [4]. A total of 96 CRFs have been identified so far [5]. HIV-1 genotypes are associated with transmission routes, and vary in epidemic size and distribution features [6]. The distribution of HIV-1 genotypes in several regions of China had been described [7–9]. However, little data is known about that in Shanghai, which is the economic, financial, trade and shipping center of mainland China. Moreover, with the development of social economy and the rapid growth of migrants, the distribution of HIV-1 genotypes may have become more diverse and complex due to HIV-1 population movements and the ongoing recombination between different HIV-1 subtypes.

In addition to genetic diversity, another consequence of high variability of HIV is its liability to develop resistance mutation to antiretroviral (ARV) drugs, including “transmitted drug resistance” (TDR), “acquired drug resistance”, and “pretreatment drug resistance (PDR)”, which can not only increase the risk of antiretroviral treatment (ART) failure, but also can lead to the spread of resistant strains, thus posing a major challenge for controlling the HIV epidemic [10]. PDR is detected in ART-naive people initiating ART or people with prior ARV drug exposure initiating or reinitiating first-line ART. However, PDR testing is not routinely performed in China as a developing country with limited conditions. So, we conducted this study to investigate the distribution of HIV-1 genotypes and prevalence of PDR in newly diagnosed HIV-1 infected patients in Shanghai China.

## Methods

### Study design and participants

A cross-sectional study was performed in Shanghai Public Health Clinical Center (SPHCC), which is the only designated hospital providing the ART and long-term follow-up for HIV/AIDS patients in Shanghai China. A total of 338 patients were enrolled in the study according to the following inclusion criteria: 1) visited SPHCC during the period from Jan 1st, 2017 to Nov 30th, 2017, and had been diagnosed with HIV/AIDS within three months before the visit; 2) had no evidence of receiving ART before the visit; 3) held the Shanghai household registration or residence permit; 4) signed the informed consent for PDR testing, and the plasma sample was successfully collected. Basic epidemiological data such as sex, age, marital status, and self-reported transmission route were recorded upon enrollment. CD4^+^T cell counts were detected by the flow cytometry. Ethical approval was obtained from the Ethics Committee of SPHCC.

### RNA extraction, nested-PCR, and sequencing of viral DNA

The plasma samples were collected and preserved in a − 80 °C freezer until analysis. Viral RNA was extracted from 140 μl plasma using QIAmp Viral RNA Mini Kit (Qiagen, Germany) according to the manufacturer protocol. Then, the target fragment of 1316 bp in the *pol* gene spanning the reverse transcriptase and protease regions was amplified using a nested polymerase chain reaction (PCR). PrimeScript™ one-step RT-PCR ver. 2.0 (TakaRa, China) was used for the cDNA synthesis and first-round PCR operation. The nested PCR was performed with Ex Taq (TaKaRa, China). The PCR products were sent to BioSune Biotechnology Co. for sequencing (Applied Biosystems, 3730XL). The PCR protocol and primers used were as described previously [8]. The primers for PCR and sequencing are listed in Table 1.

**Table 1:** List of primers for PCR and sequencing of *pol* gene in the study

Procedure	Name (direction)	Sequences(5’-3’)	Position^a^	Length of target fragment
The first round RT-PCR	MAW 26 (F)	TTGGAAATGTGGAAAGGAAGGAC	2028–2050	1513
	RT21 (R)	CTGTATTTCTGCTATTAAGTCTTTTGATGGG	3539–3509	
The second round PCR	PRO-1 (F)	CAGAGCCAACAGCCCCACCA	2147–2166	1316
	RT20 (R)	CTGCCAGTTCTAGCTCTGCTTC	3462–3441	
Sequencing	PROS3 (F)	GCCAACAGCCCCACCA	2151–2166	692
	PROC1S (R)	GCTGGGTGTGGTATTCC	2842–2826	
	RTB (F)	CCTAGTATAAACAATGAG ACAC	2946–2967	511
	RT20S3 (R)	GTTCTAGCTCTGCTTC	3456–3441	
	RTAS (F)	CTCAGATTGGTTGCAC	2524–2539	Single-Read Sequencing

^a^Nucleotide positions with reference to the HIV HXB2 strain (Genbank accession number K03455)

F -forward; R-reverse

### Identification of HIV-1 genotypes and drug-resistance mutations (DRMs)

HIV-1 genotypes were identified by phylogenetic analysis. HIV-1 pol sequences, together with reference sequences of different subtypes and CRFs, were aligned and further edited manually using the BIOEDIT version 7 (http://www.mbio.ncsu.edu/BioEdit/bioedit.html). All the subtyping reference sequences were downloaded from the Los Alamos HIV database (https://www.hiv.lanl.gov/content/sequence/HIV/mainpage.html). The phylogenetic trees were generated using the Neighbor-joining method. DRMs and resistance levels were determined based on Stanford University HIV Drug Resistance Database (HIVDB): HIVdb Program (https://hivdb.stanford.edu/hivdb/by-sequences/). The degree of drug resistance to each ARV was divided into five levels: susceptible, potential low-level resistance, low-level resistance, intermediate resistance, and high-level resistance, according to the HIVDB Genotypic Resistance Test (GRT) Interpretation System (Updated October 2018, https://hivdb.stanford.edu/assets/media/genotypic-resistance-test-interpretation-system-oct2018.57d61710.pdf).

### Statistical analysis

Data were analyzed using SPSS 22.0 (IBM Corp., Armonk, NY). Normality of data was assessed by the Kolmogorov–Smirnov test. Quantitative data with normal distribution were expressed as means ± standard deviation (x ± SD) and compared using t tests; quantitative data with skewed distribution were expressed as median (inter-quartile range, IQR) and compared using the Mann-Whitney U test. Categorical variables were expressed as frequencies and percentages and compared using the chi-square (x^2^) test or Fisher exact test. The logistic regression analysis was used to identify risk factors associated with PDR. All tests were two tailed, and *p* values < 0.05 were considered significant.

### Nucleotide sequence accession numbers

For similar scientific and ethical reasons as explained in Esbjörnsson et al. [11], only a proportion of the 317 sequences is accessible via GenBank (accession numbers, MK573428 - MK573507).

## Results

### Demographic and clinical characteristics of the participants

A total of 338 HIV-1 infected patients were enrolled in the study, and *pol* gene sequences were successfully amplified and analyzed from the plasma samples of 317 participants, including 302 (95.3%) male and 15 (4.7%) female. The median age was 33 years (IQR: 29–44 years), ranging from 20 to 79 years. Most of the participants (68.5%) were single, 28.1% were married, and 3.5% were divorced/widowed. The self-reported risk factors for HIV-1 infection were mainly homosexual contact (68.8%) and heterosexual contact (14.8%); two (0.6%) cases via intravenous drug use, and one case (0.3%) via blood transfusion; however, 49 (15.5%) patients did not know how they got infected. The median CD4 count was 275 cells/μL (IQR: 168–368 cells/μL), ranging from 1 to 1814 cells/μL. A total of 98 patients (30.9%) had a CD4 count < 200 cells/μL, 124 patients (39.1%) had a CD4 count between 200 and 350 cells/μL, and 95 patients (30.0%) had a CD4 count ≥350 cells/μL. There were no significant differences in age, sex, marital status, self-reported risk factor for HIV-1 infection, and CD4 counts among the groups divided by HIV-1 genotypes (Table 2).

**Table 2:** Demographic and clinical characteristics of the participants

Characteristics	Total (*n* = 317)	HIV-1 genotypes			*P*
		CRF01_AE (*n* = 168)	CRF07_BC (*n* = 94)	Others (*n* = 55)	
Age (years, median)	33	33	33	36	0.551
Sex (n, %)					
male	302 (95.3)	161 (95.8)	89 (94.7)	52 (94.5)	0.880
female	15 (4.7)	7 (4.2)	5 (5.3)	3 (5.5)	
Marital status (n, %)					
married	89 (28.1)	41 (24.4)	26 (27.7)	22 (40)	0.203
single	217 (68.5)	121 (72.0)	63 (67.0)	32 (58.2)	
divorced	11 (3.5)	6 (3.6)	5 (5.3)	1 (1.8)	
Self-reported risk factor (n, %)					
homosexual	218 (68.8)	121 (72.0)	65 (69.1)	32 (58.2)	0.361
heterosexual	47 (14.8)	22 (13.1)	14 (14.9)	11 (20)	
IDU	2 (0.6)	1 (0.6)	1 (1.1)	0	
Blood transfusion	1 (0.3)	0	0	1 (1.8)	
Unknown	49 (15.5)	24 (14.3)	14 (14.9)	11 (20)	
CD4 count (cells/μl, median**)**	275	270	289	266	0.085

*IDU* intravenous drug use

The differences in age and CD4 counts were tested by independent-samples Mann-Whitney U test. The differences in sex, marital status, self-reported risk factor were tested using chi-square or Fisher exact tests, if appropriate

### Distribution of HIV-1 genotypes

**Fig. 1:**
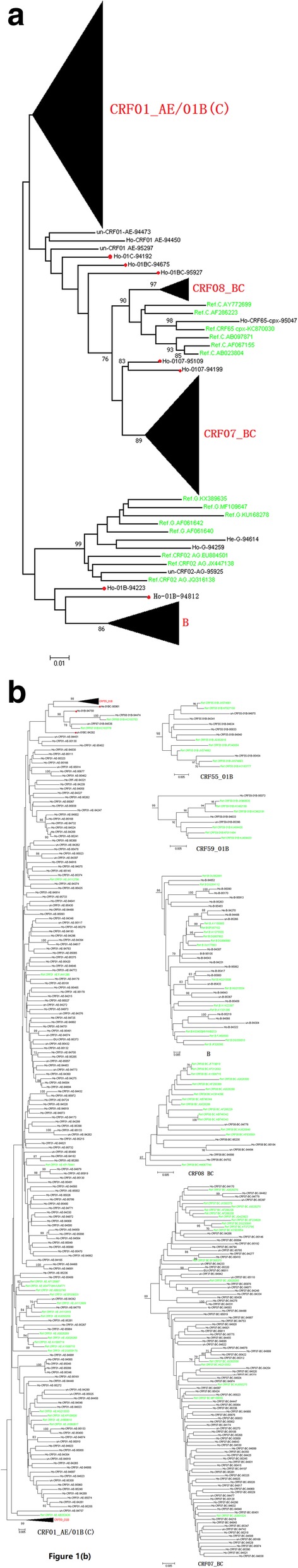
(**a** and **b**) Phylogenetic trees of HIV-1 *pol* genes were constructed using MEGA 7 based on neighbor-joining methods. **a** (total tree) shows clusters of all the sequences, whereas **b** (subtree) indicates mainly clusters of different subtypes. The strain name of the reference sequences are shown in green. The bootstrap values greater than 70% based on 1000 replicates are shown on the major branches. The scale bar indicates 0.01 for the total tree and 0.005 for the subtree. The newly identified strains are named in “transmission route-subtypes-sample code”. Ho represents homosexual; He represents heterosexual; un represents unknown; IDU represents intravenous drug use. In front of strain name, newly identified unique recombinant forms (URFs) are color-coded in red dots

**Fig. 2:**
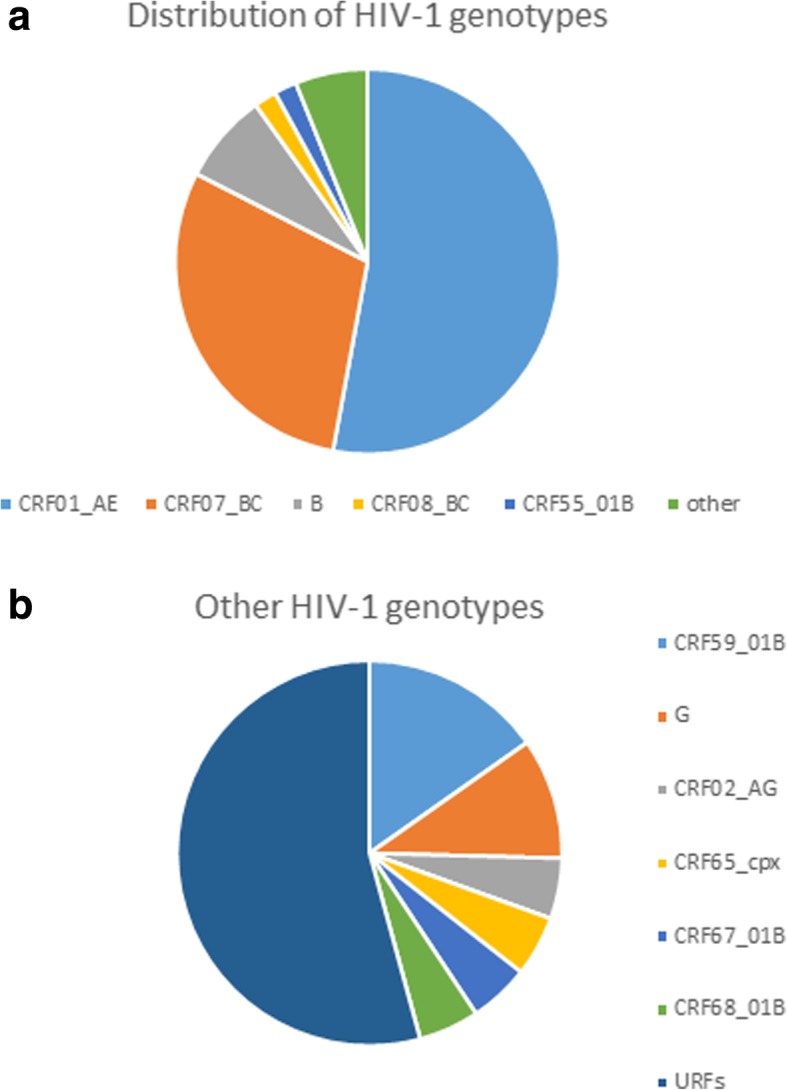
(**a** and **b**) Distribution of HIV-1 genotypes among the newly diagnosed HIV/AIDS patients in Shanghai China

Among the 317 successfully amplified samples, CRF01_AE (53.0%, 168/317) was the predominant genotype, followed by CRF07_BC (29.7%, 94/317), B (7.6%, 24/317), CRF08_BC (1.9%, 6/317), CRF55_01B (1.9%, 6/317), CRF59_01B (0.9%, 3/317), G (0.6%, 2/317). In addition, 4 strains were identified as other CRFs, including CRF02_AG (0.3%, 1/317), CRF67_01B (0.3%, 1/317), CRF68_01B (0.3%, 1/317), CRF65_cpx (0.3%, 1/317). However, another 10 (3.2%) HIV-1 strains did not cluster with any present known reference sequences, and were determined as URFs. Further analysis using REGA HIV-1 Subtyping Tool – Version 3.0 (http://dbpartners.stanford.edu:8080/RegaSubtyping/stanford-hiv/typingtool/) and HIV blast (https://www.hiv.lanl.gov/content/sequence/BASIC_BLAST/basic_blast.html), these strains were likely recombinants of CRF01_AE/B (Ho-01B-94,758, Ho-01B-94,223, Ho-01B-94,812), CRF01_AE/B/C (He-01 BC-95961, Ho-01 BC-94675, Ho-01 BC-95927, un-01 BC-94292), CRF01_AE/C (Ho-01C-94,192), and CRF01_AE/CRF07_BC (Ho-0107-94,199, Ho-0107-95,109) (Fig. 1 and Fig. 2).

### Prevalence and risk factors of PDR associated mutations

**Fig. 3:**
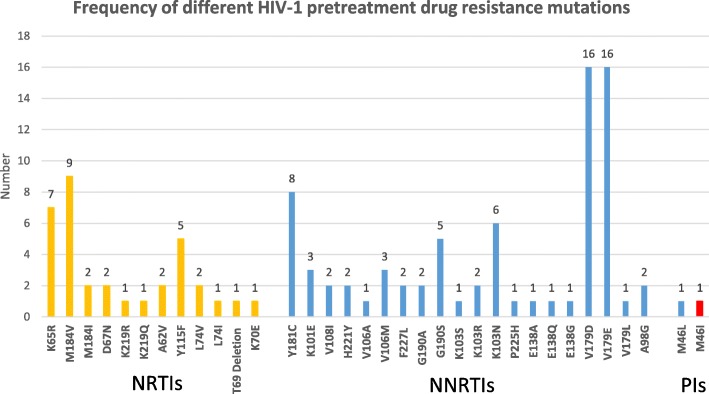
Frequency of different HIV-1 pretreatment drug resistance mutation patterns identified among 317 newly diagnosed HIV/AIDS patients in Shanghai. NRTIs: nucleoside reverse transcriptase inhibitors; NNRTIs: non-nucleoside reverse transcriptase inhibitors; PIs: protease inhibitors

The overall prevalence of PDR mutation among the 317 participants was 17.4% (55/317). Twelve nucleoside reverse transcriptase inhibitor (NRTI) resistance associated mutation patterns, 21 non-nucleoside reverse transcriptase inhibitor (NNRTI) mutation patterns, and two protease inhibitor (PI) mutation patterns were identified, respectively. The most frequent NNRTI associated mutation was V179D/E, which was observed in 10.1% (32/317) of patients, followed by Y181C (2.5%, 8/55) and K103 N (1.9%, 6/317). M184 V (2.8%, 9/317) was the most prevalent NRTI associated mutation, followed by K65R (2.2%, 7/317). The majority (72.7%, 40/55) of HIV-1 variants with PDR mutation displayed a single drug class resistance mutation. Fifteen (27.3%, 15/55) strains harbored both NRTI and NNRTI mutations. No HIV-1 strain with PDR mutations to triple classes of drugs was found in this study. Moreover, most (69.1%, 38/55) of the HIV-1 variants with mutation exhibited single mutation; two base mutation were found in 4 (7.3%) samples, and multiple (> = 3) base mutations in 13 (23.6%) samples (Fig. 3).

The multinomial logistic regression analysis for risk factors of PDR showed that the HIV-1 genotype was a potential influencing factor associated with PDR. CRF07_BC strains had a lower risk of PDR (odds ratio, 0.139; 95% CI, 0.030–0.639; *p* = 0.011) (Table 3).

**Table 3:** Multinomial logistic regression analysis for risk factors of pretreatment drug resistance

Factors	*p*	OR	95% CI for OR
age	0.385	0.984	0.950–1.020
CD4 count	0.172	0.999	0.997–1.001
HIV-1 transmission route			
homosexual	0.974	1.015	0.409–2.522
heterosexual	0.415	0.582	0.159–2.136
Marital status			
married	0.694	0.698	0.116–4.183
single	0.418	0.476	0.079–2.867
HIV-1 genotype			
CRF01_AE	0.385	0.566	0.157–2.042
CRF07_BC	0.011	0.139	0.030–0.639
CRF08_BC	0.565	1.944	0.202–18.672
CRF55_01B	0.107	4.695	0.715–30.839
B	0.477	0.564	0.117–2.730

*OR* odds ratio, *CI* confidence interval

### Resistance level to different ARV drugs (Fig. 4)

We analyzed HIV-1 resistance level to a total of 13 commonly used ARV drugs, including lamivudine (3TC), abacavir (ABC), emtricitabine (FTC), tenofovir disoproxil fumarate (TDF), zidovudine (AZT), doravirine (DOR), efavirenz (EFV), nevirapine (NVP), etravirine (ETR), rilpivirine (RPV), atazanavir/r (ATV/r), lopinavir/r (LPV/r) and darunavir/r (DRV/r). The overall prevalence of HIV-1 PDR to NNRTI (16.4%, 52/317) was much higher than that to NRTI (4.7%, 15/317) and PI (0.6%, 2/317) (X^2^ = 63.10, *p* < 0.0001). In addition, nearly half of (49.1%, 27/55) HIV-1 strains with PDR mutation exhibited the degree of potential low-level resistance to NNRTI ascribed to V179D/E.

**Fig. 4:**
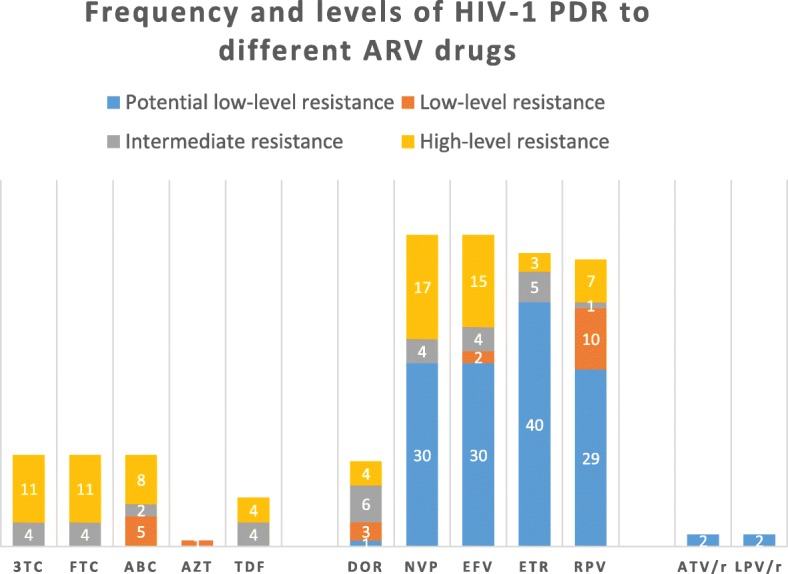
Frequency and levels of HIV-1 pretreatment drug resistance to different antiretroviral drugs among 317 newly diagnosed HIV/AIDS patients in Shanghai. 3TC: lamivudine; ABC: abacavir; FTC: emtricitabine; TDF: tenofovir disoproxil fumarate; AZT: zidovudine; EFV: efavirenz; DOR: doravirine; NVP: nevirapine; ETR: etravirine; RPV: rilpivirine; ATV: atazanavir/r; LPV: lopinavir/r

All the 15 HIV-1 strains with NRTI resistance mutations were simultaneously resistant to 3TC, ABC, and FTC. PDR frequency to AZT (0.3%, 1/317) was significantly lower than that to TDF (2.5%, 8/317) (X^2^ = 5.523, *p* = 0.019) and 3TC/ABC/FTC (4.7%, 15/317) (X^2^ = 12.567, *p* < 0.0001). There was no significantly difference between the prevalence of PDR to TDF and 3TC/ABC/FTC (X^2^ = 2.211, *p* = 0.137).

For NNRTI, DOR showed the lowest PDR frequency (4.4%, 14/317) (x^2^ = 27.522, *p* < 0.0001). The PDR frequency to EFV (16.1%, 51/317), NVP (16.1%, 51/317), ETR (15.1%, 48/317) and RPV (14.8%, 47/317) was similar (x^2^ = 0.307, *p* = 0.959). The degree of resistance was mainly at a potential low level, accounting for 0.3% (1/317), 9.5% (30/317), 9.5% (30/317), 12.6% (40/317), and 9.1% (29/317) for DOR, EFV, NVP, ETR, and RPV, respectively.

PI resistance were detected in only two samples, being simultaneously resistant to ATV/r and LPV/r at a potential low level caused by M46I/L mutation. There was no HIV-1 strain resistant to DRV/r found in our study.

## Discussion

This study showed that CRF01_AE (53.0%) was the predominant HIV-1 genotype in Shanghai, followed by CRF07_BC (29.7%) and B (7.6%). There was a high prevalence (17.4%) of PDR mutation in the newly diagnosed treatment-naive HIV/AIDS patients in Shanghai. The PDR mutation frequency to NNRTIs (16.4%) was much higher than NRTIs (4.7%) and PIs (0.6%). Moreover, about half (49.1%, 27/55) of the virus strains with mutations presented as potential low-level resistant to NNRTI ascribed to V179D/E.

HIV-1 is characterized by its vast genetic variability caused by high-rate but error-prone replication and recombination of the virus. The global distribution of HIV-1genotypes is highly heterogeneous and varies geographically [6]. Furthermore, the HIV-1 epidemic may evolve along with the change in transmission risk behaviours, lifestyle and the patterns of human mobility. Various aspects of HIV infection may be affected by its diversity, including disease progression [12], response to ART, transmission routes, vaccine development, immune response and escape, etc. [6]. It is of great significance to know the epidemiological characteristics of AIDS in a certain area, so as to better control the epidemic. Our study found a total of 11 HIV-1 genotypes and 10 HIV-1 strains of URFs in 317 newly diagnosed HIV/AIDS patients in 2017, indicating that the distribution of HIV-1 genotypes in Shanghai is diverse and complex. The results were similar to that of the study conducted in Jiangsu province (a close neighbor of Shanghai), showing that the prevalence of genotype CRF01_AE, CRF07_BC, and B were 60.06, 22.29, and 5.88%, respectively [13]. Similarly, the surveys carried out in Hebei, Fujian, and regions across China also reported that CRF01_AE, CRF07_BC, and B were the predominant subtypes [9, 14, 15]. However, some other studies displayed different results. For example, a study performed in 205 HIV (+) blood donors from five Chinese blood centers showed that CRF07_BC (61.5%) was the most prevalent, followed by CRF01_AE (20%) and CRF08_BC (8.3%) [16]. Besides, a recent investigation on HIV-1 genetic characteristics in Yunnan province revealed that CRF08_BC (47.4%) was the most common, followed by URFs (18.2%), CRF01_AE (15.8%) and CRF07_BC (14.4%) [17]. The reasons for the discrepancy may be attributed to the difference in the transmission routes. According to the national official report, heterosexual contact has become the predominant risk factor for HIV epidemic in China [2]. While, in our study, homosexual contact accounted for 68.8% of HIV transmission factors for Shanghai cases. Chen’s study revealed that CRF07_BC was increasing among men who have sex with men (MSM) in Fujian; our findings also indicated that CRF07_BC was one of the most prevalent genotypes among MSM in Shanghai [9].

Another serious consequence of HIV-1 high variability is drug resistance - an emerging threat to epidemic control, which can cause treatment failure and further spread of drug resistant HIV. According to WHO surveys of PDR between 2014 and 2016, seven Latin American and African countries estimated a prevalence of PDR greater than 10% in adults initiating ART [18]. Our study in Shanghai also showed a high prevalence of PDR among the newly diagnosed HIV-1 infected patients in 2017, which is higher than that in Hebei, Beijing and Yunnan province [8, 19, 20]. ART has been widely used in China since 2006, when the government’s “Four Free and One Care” policy - free antiretroviral drugs to AIDS patients who are rural residents or people without insurance living in urban areas; free voluntary counselling and testing; free drugs to HIV-infected pregnant women to prevent mother-to-child transmission, and HIV testing of newborn babies; free schooling for AIDS orphans; care and economic assistance to the households of people living with HIV/AIDS - for AIDS control was introduced. Because of the limited availability of drugs in China, the regimen composed of TDF, 3TC and EFV is currently the most commonly used free first-line therapy. According to our study, PDR mutation frequency to NNRTIs (16.4%) was much higher than NRTIs (4.7%) and PIs (0.6%) in Shanghai, and the above three kinds of drugs exhibited a PDR mutation frequency of 2.5% (TDF), 4.7% (3TC) and 16.1% (EFV), respectively, which highlights the importance of routinely PDR testing. WHO’s new recommendations on the public health response to PDR indicate that in countries where population-levels of PDR to NNRTI reach the threshold of 10%, a change in the first-line ART regimen (from NNRTI-based to non-NNRTI based, such as integrase inhibitors) should be urgently considered [21]. Therefore, PI/integrase inhibitor-containing regimen may be preferred initial therapy for the newly diagnosed HIV/AIDS patients in Shanghai.

However, it should be noted that for about half (49.1%) of HIV-1 strains with drug resistant mutations, the degree of resistance was classified as potential low-level due to the single V179D/E mutation, which is selected by NNRTI and contribute low-levels reductions in susceptibility to each of the NNRTIs. However, whether this single mutation can lead to clinical treatment failure needs to be further studied and confirmed. The trend of increasing V179D/E mutation in genotype CRF01_AE among MSM population was also reported in other study [22].

In addition, our study showed that HIV-1 genotype was a potential influencing factor of PDR. CRF07_BC strains had a lower risk for PDR. However, another study revealed that genotype C was associated with high risk for resistance [8]. The reason for this difference need further study to demonstrate. However, due to the small sample size further research in a larger population is necessary to confirm that.

We have to point out that there are some limitations of this study. First, sample selection bias may exist, because the newly diagnosed AIDS patients who didn’t sign the consent form for the pretreatment HIV-1 drug resistance testing were not included in the study. The majority of the participants were male and MSM. Second, our analysis about PDR concentrated only on NRTI, NNRTI and PI, not containing integrase inhibitors, which are a newer kind of ARV drugs. It is expected that resistance to integrase inhibitors will invariably emerge with their increasingly widely used in ART-naive and ART-experienced patients. Third, the participants enrolled in the study were newly diagnosed with HIV-1 infection in 2017. However, many patients didn’t know when they had got HIV-1 infection. Some patients might have been infected for a relatively long time. Therefore, the finding may not really reflect the current HIV epidemic in Shanghai.

## Conclusions

In summary, our study provides the knowledge of molecular epidemiological characteristics of HIV-1 and the prevalence of PDR among the newly diagnosed AIDS patients in Shanghai in 2017. The distribution of HIV-1 genotypes in Shanghai is diverse and complex, with CRF01_AE and CRF07_BC as the predominant genotypes. The high PDR mutation frequency in ART-naive patients highlights the significance of routine testing of pretreatment HIV-1 drug resistance, and non-NNRTI-containing regimen, such as PI/integrase inhibitor-based regimen may be the preferred initial therapy for this population by reason of high prevalence of PDR to NNRTI.

## Abbreviations

3TC: Lamivudine
ABC: Abacavir
ART: Antiretroviral treatment
ARV: Antiretroviral
ATV/r: Atazanavir/r
AZT: Zidovudine
CRF: Circulating recombinant form
DOR: Doravirine
DRMs: Drug-resistance mutations
EFV: Efavirenz
ETR: Etravirine
FTC: Emtricitabine
IDU: Intravenous drug use
IQR: Inter-quartile range
LPV/r: Lopinavir/r
NNRTI: Non-nucleoside reverse transcriptase inhibitor
NRTI: Nucleoside reverse transcriptase inhibitor
NVP: Nevirapine
PCR: Polymerase chain reaction
PDR: Pretreatment drug resistance
PI: Protease inhibitor
RPV: Rilpivirine
SPHCC: Shanghai Public Health Clinical Center
TDF: Tenofovir disoproxil fumarate
TDR: Transmitted drug resistance
URFs: Unique recombinant forms
